# Efficacy and safety of immune checkpoint inhibitors combined with TKIs for recurrent or metastatic cervical cancer with PIK3CA mutation

**DOI:** 10.1371/journal.pone.0350571

**Published:** 2026-09-29

**Authors:** Lele Chang, Yaxin Kang, Mingxuan Zhu, Jing Liu, Chunyan Lan, Qin Xu

**Affiliations:** 1 Department of Gynecology, Clinical Oncology School of Fujian Medical University, Fujian Cancer Hospital, Fuzhou, Fujian, P. R. China; 2 Department of Gynecologic Oncology, Sun Yat-sen University Cancer Centre, Guangzhou, Guangdong, P. R. China; 3 State Key Laboratory of Oncology in South China, Collaborative Innovation Centre for Cancer Medicine, Guangzhou, Guangdong, P. R. China; 4 Fujian Key Laboratory of Advanced Technology for Cancer Screening and Early Diagnosis, Fujian Cancer Hospital, Fuzhou, Fujian, China; University of Wisconsin, UNITED STATES OF AMERICA

## Abstract

**Background:**

PIK3CA mutations are among the most common driver mutations in cervical cancer (CC), yet therapeutic strategies targeting PIK3CA mutations are still under investigation. This study aims to evaluate the efficacy and safety of immune checkpoint inhibitors (ICIs) combined with tyrosine kinase inhibitors (TKIs) in patients with PIK3CA-mutated recurrent or metastatic (R/M) CC.

**Methods:**

This study was based on two prospective clinical trials (ChiCTR1900023015 and the CLAP study) and retrospective data from Fujian Cancer Hospital, comparing ICIs combined with TKIs with physician’s-choice Non-ICI/TKI conventional treatments in PIK3CA-mutated R/M CC. The primary endpoint was objective response rate (ORR), and secondary endpoints included disease control rate (DCR), progression-free survival (PFS), overall survival (OS), and safety.

**Results:**

A total of 55 patients were included, with 27 in the ICIs plus TKIs treatment group and 28 in the Non-ICI/TKI conventional treatment group. The ORR in the ICIs plus TKIs group was 85.19% (95% CI, 67.52%−94.08%), significantly higher than 28.57% (95% CI, 15.25%−47.06%) in the Non-ICI/TKI conventional treatment group (P < 0.001). The PFS in the ICIs plus TKIs group was 21.03 months, significantly longer than 9.13 months in the Non-ICI/TKI conventional treatment group (HR, 0.49; 95% CI, 0.26–0.94, P = 0.033). Although the OS difference was not statistically significant (HR, 0.65; 95% CI, 0.31–1.34, P = 0.24), the ICIs plus TKIs group showed superior clinical efficacy. The incidence of hypertension (P < 0.001), rash (P = 0.004), hypothyroidism (P < 0.001), proteinuria (P = 0.010), and palmoplantar erythrodysesthesia syndrome (P = 0.023) was higher in the ICIs plus TKIs group compared to the Non-ICI/TKI conventional treatment group. The observed adverse events were generally manageable. Although grade ≥3 adverse events occurred, no grade ≥3 immune-related adverse events or treatment-related deaths were observed.

**Conclusion:**

ICIs plus TKIs demonstrated significant efficacy in treating PIK3CA-mutated R/M CC. Although there were some safety concerns, the adverse reactions were manageable, no grade ≥3 immune-related adverse events or treatment-related deaths were observed.

## Introduction

Cervical cancer (CC) is the fourth leading cause of cancer-related deaths in women worldwide, with an estimated 604,000 new cases in 2020 [1]. In China, CC ranks second in terms of incidence among malignant tumors of the female reproductive system [2]. About 20%−30% of patients experience recurrence or metastasis (R/M) after treatment, and treatment options are limited for those with advanced disease, leading to poor prognosis [3]. However, advances in cancer treatment have improved the prognosis of CC patients. Despite the best therapeutic interventions, patients with R/M CC have a poor prognosis, with a 5-year survival rate of only 17% [3]. With the advent of the immunotherapy era, immune-based drugs have brought new hope to cancer patients. Clinical trials such as KEYNOTE-028 [4] and KEYNOTE-158 [5] have demonstrated the efficacy and safety of pembrolizumab in patients with R/M CC who experience disease progression after chemotherapy. The results of the KEYNOTE-158 study showed that pembrolizumab monotherapy had a median progression-free survival (PFS) of 2.1 months, a median overall survival (OS) of 11 months, and an objective response rate (ORR) of 14.3% in PD-L1-positive tumors [5]. Although most CC express PD-L1, only a small proportion of PD-L1-positive patients benefit from immunotherapy. Therefore, there is an urgent need for combination therapies to enhance the antitumor effects in CC patients [6].

The GOG 240 trial showed that bevacizumab combined with chemotherapy significantly improved OS and ORR in first-line treatment for advanced CC [7], indicating that anti-angiogenic therapy may offer clinical benefits for patients with R/M CC. In recent years, several clinical trials have confirmed that the combination of anti-angiogenic drugs with immunotherapy yields significant therapeutic efficacy in various malignancies [8,9]. Combining anti-angiogenic agents with immunotherapy can normalize the abnormal tumor vasculature, increase the infiltration of immune effector T cells, and promote T cell distribution within the tumor, effectively converting the immune-suppressive tumor microenvironment into an immune-enhancing one [10]. Our previous clinical studies (ChiCTR1900023015) [11] and the CLAP study (NCT03816553) [12] have shown that immune checkpoint inhibitors (ICIs) combined with tyrosine kinase inhibitors (TKIs) has significant therapeutic effects in R/M CC, significantly improving patients’ ORR and PFS.

Phosphoinositide 3-kinase catalytic subunit alpha (PIK3CA) is one of the most frequently mutated oncogenes in cancer [13]. Most mutations occur in hotspot codons H1047, E542, E545, and N345. In our previous biomarker analysis of clinical studies and the CLAP trial, we found that PIK3CA was a common mutated gene (31.7% and 44.0%, respectively) and that it was associated with the efficacy of ICIs and TKIs combination therapy. These findings motivated a focused exploratory analysis of clinical outcomes among patients with PIK3CA-altered R/M cervical cancer [11,12]. In a retrospective analysis of NGS data from 98 CC patients, we found that the frequency of PIK3CA mutations was 40.0%, which further supports these findings [14]. However, there is insufficient evidence to support the safety and efficacy of ICIs combined with TKIs in patients with R/M CC harboring PIK3CA mutations, compared to Non-ICI/TKI conventional treatment regimens.

This exploratory study aimed to evaluate the safety and efficacy of ICIs combined with TKIs compared with physician’s-choice non-ICI/TKI treatments among patients with PIK3CA-altered R/M cervical cancer. This research seeks to provide a new treatment option for R/M CC patients with PIK3CA mutations.

## Materials and Methods

### Inclusion and exclusion criteria

This study includes patients with R/M CC from two prospective clinical trials: ChiCTR1900023015 [11] and the CLAP study (NCT03816553). Additionally, we retrospectively collected data from patients treated with conventional therapies for R/M CC at Fujian Cancer Hospital between October 1, 2017, and March 31, 2024, as a control cohort. The inclusion criteria were as follows: 1) Pathologically diagnosed as cervical cancer; 2) Patients with measurable lesions in R/M CC; 3) At least one prior treatment regimen; 4) Eastern Cooperative Oncology Group (ECOG) performance status (PS) of 0−1; 5) Next-generation sequencing (NGS) results confirming PIK3CA mutations in CC. Exclusion criteria included: 1) Any other active malignancy within the past 5 years; 2) No measurable target lesions. This study was approved by the Ethics Committee of Fujian Cancer Hospital (approval number: 2019-074-02) and conducted in accordance with the Declaration of Helsinki.

### Treatment regimens

We included two prospective clinical trials as the immunotherapy combined with TKIs cohort: 1) ChiCTR1900023015 Study: Patients received 200 mg of sintilimab intravenously on day 1 every 3 weeks, and 10 mg of anlotinib orally once daily from days 1–14 of each cycle. Treatment continued until disease progression (PD), treatment intolerance, death, or initiation of a new anti-cancer therapy; 2) CLAP Study: Patients received 200 mg of camrelizumab intravenously every two weeks and 250 mg of apatinib orally once daily, with a 4-week treatment cycle, for up to two years. Treatment continued until disease progression, unacceptable toxicity, or withdrawal of consent. The comparator cohort was defined as a physician’s-choice non-ICI/TKI conventional treatment cohort (non-ICI/TKI conventional treatment). This cohort retrospectively included patients with PIK3CA-mutated R/M CC who did not receive ICIs combined with TKIs and were treated at Fujian Cancer Hospital during the same study period. Treatment regimens in this cohort were selected by the treating physicians according to disease status, prior therapy, performance status, organ function, patient preference, drug availability, and real-world clinical practice. These regimens included radiotherapy alone, chemotherapy alone, targeted therapy alone, concurrent chemoradiotherapy, targeted therapy combined with radiotherapy or chemotherapy, and other individualized treatments.

### Study methods

#### Clinical information.

We collected clinical and pathological data, including age, body mass index (BMI), ECOG PS, median time from initial cancer diagnosis to study enrollment, target lesion size, International Federation of Gynecology and Obstetrics (FIGO) stage (2018) at initial diagnosis, histology, R/M information, prior treatment regimens, PIK3CA mutation sites and types, programmed cell death ligand 1(PD-L1) expression, tumor mutational burden (TMB), treatment efficacy results, disease progression time and causes, treatment-related adverse events, and survival status at the last follow-up, including date of death and cause of death.

### Treatment response evaluation

We assessed treatment responses based on the response evaluation criteria in solid tumors (RECIST 1.1). Treatment responses were categorized into complete response (CR), partial response (PR), stable disease (SD), PD. Treatment toxicity was graded according to the National Cancer Institute’s Common Terminology Criteria for Adverse Events (CTCAEs) version 4.03.

### Study endpoints

The primary endpoint of this study is the objective response rate (ORR), defined as the proportion of patients achieving CR or PR. Secondary endpoints include PFS (time from first treatment to PD or death from any cause), OS (time from first treatment to death from any cause), disease control rate (DCR, proportion of patients achieving CR, PR, or SD), and safety and tolerability. ORR and DCR were calculated in the full analysis population, defined as all eligible patients included in this study. Patients without post-baseline response evaluation were included in the denominator and were counted as non-responders for ORR and as not achieving disease control for DCR. The final follow-up date for this study was September 30, 2024.

### Biomarker analysis

We evaluated the expression level of PD-L1 in tumor samples by immunohistochemistry and measured the Combined Positive Score (CPS). CPS is defined as the proportion of PD-L1–positive cells among all viable tumor cells. NGS was used to assess the TMB, which is defined as the total number of somatic mutations (including base substitutions, insertions, and deletions) detected per million bases.

### Consent for publication

All the authors agreed to publish this work.

### Statistical analysis

Continuous variables were summarized as means (standard deviation), medians (interquartile range), and ranges (minimum, maximum), and group comparisons were performed using non-parametric tests. Chi-square tests and Fisher’s exact tests were used to assess differences between categorical variables. Descriptive statistics for categorical variables were presented as frequencies (percentages). Kaplan-Meier methods were used to generate survival curves, and the log-rank test was employed to compare survival rates between groups. A two-sided P-value of <0.05 was considered statistically significant. All statistical analyses were performed using R software version 4.3.2. Between-group risk ratios (RRs) with two-sided 95% confidence intervals (CIs) were calculated for ORR and DCR; group-specific response-rate CIs were estimated using the Wilson score method. Univariable Cox proportional-hazards models with treatment group as the sole covariate were used to estimate hazard ratios (HRs) and 95% CIs for PFS and OS.

Based on two prospective clinical trials and a retrospective cohort, no prospective sample size calculation was performed before patient inclusion in this study. A post hoc sample size justification was therefore performed based on the primary endpoint of ORR. The observed ORR was 85.19% in the ICIs combined with TKIs group and 28.57% in the Non-ICI/TKI conventional treatment group, with a total sample size of 55 patients. Using a two-sided α of 0.05 and Fisher’s exact test for two independent proportions, the achieved power for detecting the observed ORR difference was approximately 99.3%, corresponding to a β of approximately 0.7%.

## Results

### Patient characteristics

Between October 1, 2017, and March 31, 2024, a total of 55 patients were included in this study, with 27 patients in the ICIs combined with TKIs group and 28 patients in the non-ICI/TKI conventional treatment cohort. Most baseline characteristics were comparable between the two groups, including age, BMI, ECOG PS, time from initial cancer diagnosis to study enrollment, target lesion size, FIGO stage (2018) at initial diagnosis, histological type, local recurrence, liver metastasis, distant lymph node metastasis, other metastatic sites, previous radiotherapy, previous radical surgical treatment, previous platinum exposure, previous paclitaxel exposure, PIK3CA mutation type and mutation site, PD-L1 expression level, and tumor mutational burden (P ≥ 0.05). However, lung metastases were more frequent in the ICIs combined with TKIs group than in the Non-ICI/TKI conventional treatment cohort (51.85% vs. 17.86%, P = 0.008), and the number of previous systemic therapies differed significantly between the two groups (P = 0.012). The average age of the entire cohort was 56 years. All patients had an ECOG PS of 0–1. Table 1 summarizes the detailed clinical and pathological characteristics of the two patient groups.

**Table 1:** Patient demographics and baseline characteristics.

Characteristic	Group			p-value
	Overall (N = 55)	Non-ICI/TKI conventional treatment (N = 28)	ICIs combined with TKIs (N = 27)	
**Age**				0.062
Mean ± SD	56 ± 7.6	58 ± 6.6	54 ± 8.2	
Median (IQR)	56 (53, 62)	59 (55, 62)	55 (51, 59)	
Range	35, 74	43, 74	35, 72	
**BMI**				0.070
Mean ± SD	22.34 ± 4.118	21.48 ± 2.832	23.23 ± 5.027	
Median (IQR)	21.70 (20.12, 24.29)	21.08 (19.50, 23.44)	22.67 (20.91, 24.98)	
Range	13.97, 42.54	15.62, 28.54	13.97, 42.54	
**ECOG PS**				0.236
0	2 (3.64%)	0 (0.00%)	2 (7.41%)	
1	53 (96.36%)	28 (100.00%)	25 (92.59%)	
**Time from initial cancer diagnosis to study enrollment (months)**				0.516
Mean ± SD	22.09 ± 37.655	25.96 ± 46.142	18.07 ± 26.478	
Median (IQR)	12.00 (3.00, 24.50)	12.50 (3.75, 26.75)	11.00 (0.50, 23.50)	
Range	0.00, 240.00	0.00, 240.00	0.00, 128.00	
**Target lesion size (mm)**				0.050
Mean ± SD	35.33 ± 20.092	29.39 ± 13.964	41.48 ± 23.636	
Median (IQR)	30.00 (22.00, 42.50)	28.00 (18.50, 35.25)	34.00 (26.50, 46.50)	
Range	6.00, 91.00	6.00, 63.00	13.00, 91.00	
**FIGO stage (2018) at initial diagnosis**				0.504
IB	4 (7.27%)	1 (3.57%)	3 (11.11%)	
IIA	11 (20.00%)	6 (21.43%)	5 (18.52%)	
IIB	12 (21.82%)	7 (25.00%)	5 (18.52%)	
IIIA	2 (3.64%)	2 (7.14%)	0 (0.00%)	
IIIB	8 (14.55%)	3 (10.71%)	5 (18.52%)	
IIIC	7 (12.73%)	2 (7.14%)	5 (18.52%)	
IVB	6 (10.91%)	3 (10.71%)	3 (11.11%)	
Unknown	5 (9.09%)	4 (14.29%)	1 (3.70%)	
**Histology**				>0.999
Squamous cell carcinoma	50 (90.91%)	25 (89.29%)	25 (92.59%)	
Adenocarcinoma	5 (9.09%)	3 (10.71%)	2 (7.41%)	
**Local recurrence**				0.246
No	41 (74.55%)	19 (67.86%)	22 (81.48%)	
Yes	14 (25.45%)	9 (32.14%)	5 (18.52%)	
**Liver metastases**				0.729
No	46 (83.64%)	24 (85.71%)	22 (81.48%)	
Yes	9 (16.36%)	4 (14.29%)	5 (18.52%)	
**Lung metastases**				0.008
No	36 (65.45%)	23 (82.14%)	13 (48.15%)	
Yes	19 (34.55%)	5 (17.86%)	14 (51.85%)	
**Distant lymph nodes metastases**				0.452
No	19 (34.55%)	11 (39.29%)	8 (29.63%)	
Yes	36 (65.45%)	17 (60.71%)	19 (70.37%)	
**Other sites metastases**				0.864
No	34 (61.82%)	17 (60.71%)	17 (62.96%)	
Yes	21 (38.18%)	11 (39.29%)	10 (37.04%)	
**Previous radiotherapy**				>0.999
No	6 (10.91%)	3 (10.71%)	3 (11.11%)	
Yes	49 (89.09%)	25 (89.29%)	24 (88.89%)	
**Previous radical surgical treatment**				0.508
No	35 (63.64%)	19 (67.86%)	16 (59.26%)	
Yes	20 (36.36%)	9 (32.14%)	11 (40.74%)	
**Previous platinum**				0.422
No	7 (12.73%)	5 (17.86%)	2 (7.41%)	
Yes	48 (87.27%)	23 (82.14%)	25 (92.59%)	
**Previous paclitaxel**				0.561
No	12 (21.82%)	7 (25.00%)	5 (18.52%)	
Yes	43 (78.18%)	21 (75.00%)	22 (81.48%)	
**Previous antivascular targeted therapy**				0.051
No	48 (87.27%)	27 (96.43%)	21 (77.78%)	
Yes	7 (12.73%)	1 (3.57%)	6 (22.22%)	
**Number of previous systemic therapies**				0.012
1	31 (56.36%)	21 (75.00%)	10 (37.04%)	
2	17 (30.91%)	4 (14.29%)	13 (48.15%)	
≥3	7 (12.73%)	3 (10.71%)	4 (14.81%)	
**PIK3CA mutation types**				0.193
Gene amplification	5 (9.09%)	1 (3.57%)	4 (14.81%)	
Missense mutation	50 (90.91%)	27 (96.43%)	23 (85.19%)	
**PIK3CA mutation sites**				0.703
p.E542/545K	36 (65.45%)	19 (67.86%)	17 (62.96%)	
Others	19 (34.55%)	9 (32.14%)	10 (37.04%)	
**PD-L1(CPS)**				>0.999
≤1	8 (14.55%)	4 (14.29%)	4 (14.81%)	
1-10	18 (32.73%)	9 (32.14%)	9 (33.33%)	
≥10	29 (52.73%)	15 (53.57%)	14 (51.85%)	
**TMB**				0.069
<5	18 (32.73%)	6 (21.43%)	12 (44.44%)	
≥5	37 (67.27%)	22 (78.57%)	15 (55.56%)	

BMI: Body Mass Index; ECOG PS: Eastern Cooperative Oncology Group Performance Status; FIGO: International Federation of Gynecology and Obstetrics; TMB: Tumor Mutational Burden; ICIs: Immune Checkpoint Inhibitors; TKIs: Tyrosine Kinase Inhibitors; CPS: Combined Positive Score. P-values for continuous variables were calculated using the Wilcoxon rank-sum test. P-values for categorical variables were calculated using Pearson’s chi-square test or Fisher’s exact test, as appropriate.

### Treatment response

All patients received at least one cycle of treatment and were included in the full analysis population. In the Non-ICI/TKI conventional treatment cohort, 0 patients achieved CR, 8 patients (28.57%) achieved PR, 7 patients (25.00%) had SD, 11 patients (39.29%) experienced PD, and 2 patients (7.14%) were not evaluable for response. In the ICIs combined with TKIs group, 1 patient (3.70%) achieved CR, 22 patients (81.48%) achieved PR, 1 patient (3.70%) had SD, 2 patients (7.41%) experienced PD, and 1 patient (3.70%) was not evaluable for response. In the full analysis population, the ICIs combined with TKIs group showed a higher ORR than the Non-ICI/TKI conventional treatment cohort [85.19% (95% CI, 67.52%−94.08%) vs. 28.57% (95% CI, 15.25%−47.06%), P < 0.001]. The DCR was also higher in the ICIs combined with TKIs group than in the Non-ICI/TKI conventional treatment cohort [88.89% (95% CI, 71.94%−96.15%) vs. 53.57% (95% CI, 35.81%−70.47%), P = 0.004] (Table 2). Further subgroup analysis revealed that there was no significant difference in treatment efficacy between the ICI plus anlotinib group and the ICI plus apatinib group (P = 0.096) (S1 Table). The between-group effect estimates were RR 2.98 (95% CI, 1.63–5.47) for ORR and RR 1.66 (95% CI, 1.15–2.40) for DCR, comparing the ICIs combined with TKIs group with the Non-ICI/TKI conventional treatment cohort.

**Table 2:** Treatment efficacy between conventional treatment group and ICIs combined with TKIs group.

Efficacy	Group			p-value/ RR (95% CI)
	Overall (N = 55)	Non-ICI/TKI conventional treatment (N = 28)	ICIs combined with TKIs (N = 27)	
**Response Evaluation**				<0.001
**CR**	1 (1.82%)	0 (0.00%)	1 (3.70%)	
**PR**	30 (54.55%)	8 (28.57%)	22 (81.48%)	
**SD**	8 (14.55%)	7 (25.00%)	1 (3.70%)	
**PD**	13 (23.64%)	11 (39.29%)	2 (7.41%)	
**Unknown**	3 (5.45%)	2 (7.14%)	1 (3.70%)	
**ORR**	31 (56.36%) [95% CI, 43.27%−68.63%]	8 (28.57%) [95% CI, 15.25%−47.06%]	23 (85.19%) [95% CI, 67.52%−94.08%]	<0.001; RR 2.98 (1.63–5.47)
**DCR**	39 (70.91%) [95% CI, 57.86%−81.23%]	15 (53.57%) [95% CI, 35.81%−70.47%]	24 (88.89%) [95% CI, 71.94%−96.15%]	0.004; RR 1.66 (1.15–2.40)

CR: Complete Response; PR: Partial Response; SD: Stable Disease; PD: Progressive Disease; ORR: Objective Response Rate; DCR: Disease Control Rate; ICIs: Immune Checkpoint Inhibitors; TKIs: Tyrosine Kinase Inhibitors. P-values were calculated using Fisher’s exact test for comparisons with small expected cell counts; otherwise, Pearson’s chi-square test was used. ORR and DCR were calculated using all included patients as the denominator. Patients with unknown or non-evaluable response were included in the denominator and counted as non-responders for ORR and as not achieving disease control for DCR. Between-group RRs compare ICIs combined with TKIs with Non-ICI/TKI conventional treatment; 95% CIs for ORR and DCR were calculated using the Wilson score method for group-specific rates and the log risk-ratio method for between-group RRs.

### PFS and OS of the two groups

In the Non-ICI/TKI conventional treatment group, the median PFS was 9.13 months (95% CI, 6.53–19.30). In the ICIs combined with TKIs group, the median PFS was 21.03 months (95% CI, 11.93-not reached), which was significantly better than the Non-ICI/TKI conventional treatment group (P = 0.033) (Fig 1A). For OS, the median OS in the Non-ICI/TKI conventional treatment group was 22.9 months (95% CI, 14.70-not reached), whereas in the ICIs combined with TKIs group, the median OS was 36.93 months (95% CI, 23.67-not reached). Although the OS was longer in the ICIs combined with TKIs group, the difference did not reach statistical significance (P = 0.240) (Fig 1B). Subgroup analysis of patients in the ICIs combined with TKIs group revealed no significant differences in OS and PFS between patients in the sintilimab plus anlotinib group (ChiCTR1900023015 study) and those in the camrelizumab plus apatinib group (CLAP study, NCT03816553) (P = 0.68 and P = 0.73, respectively) (Supplementary Figures 1A and 1B). The univariable Cox model estimated an HR for progression or death of 0.49 (95% CI, 0.26–0.94; P = 0.033). There were 38 PFS events overall (15/27 [55.56%] in the ICIs combined with TKIs group and 23/28 [82.14%] in the Non-ICI/TKI conventional treatment group). For OS, the univariable Cox model estimated an HR for death of 0.65 (95% CI, 0.31–1.34; P = 0.240), with 30 deaths overall (13/27 [48.15%] and 17/28 [60.71%], respectively).

**Fig 1:**
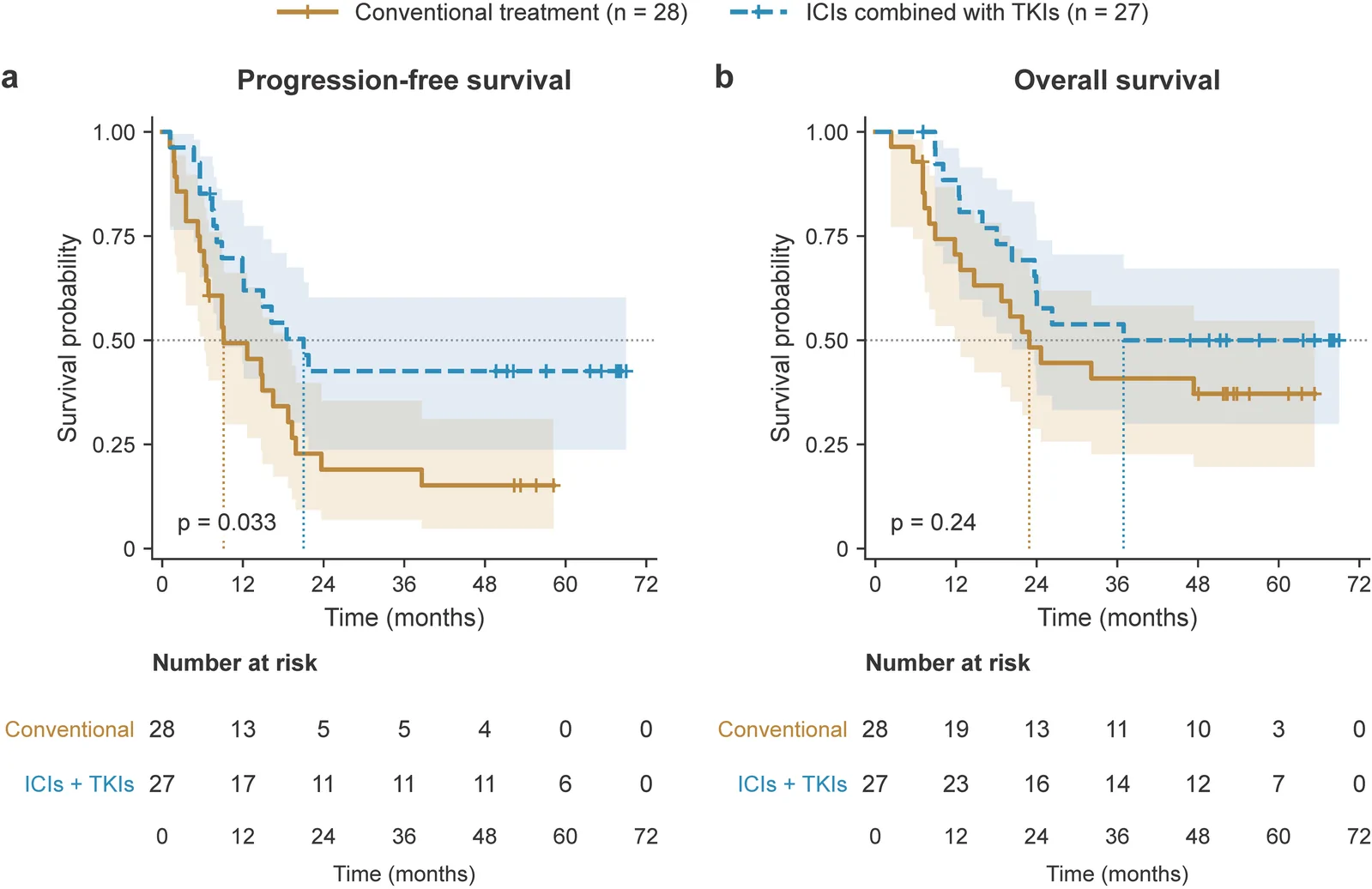
The progression-free survival (PFS) (A) and overall survival (OS) (B) survival curves comparing the Non-ICI/TKI conventional treatment group with the immune checkpoint inhibitors (ICIs) combined with tyrosine kinase inhibitors (TKIs) group.

### TRAEs

Overall, there were no significant differences in the incidence of TRAEs between the ICIs combined with TKIs group and Non-ICI/TKI conventional treatment group. However, patients in the ICIs combined with TKIs group were more likely to experience hypertension (P < 0.001), rash (P = 0.004), hypothyroidism (P < 0.001), proteinuria (P = 0.010), and palmar-plantar erythrodysesthesia syndrome (P = 0.023). Grade ≥3 adverse events occurred in both groups, but their incidence did not differ significantly between the groups. No grade ≥3 immune-related adverse events or treatment-related deaths were reported. Anemia was the most common any-grade adverse event in the ICIs plus TKIs group (55.56%), whereas anemia and leukopenia were jointly the most common adverse events in the Non-ICI/TKI conventional treatment group (53.57%). Proteinuria was significantly more frequent in the ICIs plus TKIs group than in the conventional-treatment group (37.04% vs. 7.14%; P = 0.010). In the ICIs combined with TKIs group, one patient developed immune-related pneumonitis, and one patient developed immune-related myocarditis. Detailed information on adverse events is provided in Table 3.

**Table 3:** Treatment-related adverse events in the total treated patients.

	Any grade				Grade ≥ 3			
Treatment-related adverse events	Group			p-value	Group			
	Overall	Non-ICI/TKI conventional treatment	ICIs combined with TKIs		Overall	Non-ICI/TKI conventional treatment	ICIs combined with TKIs	p-value
**Anemia**	30 (54.55%)	15 (53.57%)	15 (55.56%)	0.883	11 (20.00%)	5 (17.86%)	6 (22.22%)	0.746
**Leukopenia**	26 (47.27%)	15 (53.57%)	11 (40.74%)	0.341	4 (7.27%)	3 (10.71%)	1 (3.70%)	0.611
**Neutropenia**	24 (43.64%)	13 (46.43%)	11 (40.74%)	0.671	5 (9.10%)	4 (14.29%)	1 (3.70%)	0.352
**Thrombocytopenia**	14 (25.45%)	10 (35.71%)	4 (14.81%)	0.075	5 (9.10%)	3 (10.71%)	2 (7.41%)	1.000
**Increased aspartate aminotransferase**	17 (30.91%)	7 (25.00%)	10 (37.04%)	0.334	2 (3.64%)	1 (3.57%)	1 (3.70%)	1.000
**Increased alanine aminotransferase**	17 (30.91%)	8 (28.57%)	9 (33.33%)	0.702	2 (3.64%)	1 (3.57%)	1 (3.70%)	1.000
**Hypertension**	14 (25.45%)	1 (3.57%)	13 (48.15%)	<0.001	3 (5.45%)	0 (0.00%)	3 (11.11%)	0.111
**Hypercholesterolemia**	6 (10.91%)	2 (7.14%)	4 (14.81%)	0.422	0 (0.00%)	0 (0.00%)	0 (0.00%)	–
**Hypertriglyceridemia**	6 (10.91%)	2 (7.14%)	4 (14.81%)	0.422	0 (0.00%)	0 (0.00%)	0 (0.00%)	–
**Hypoproteinemia**	11 (20.00%)	8 (28.57%)	3 (11.11%)	0.106	1 (1.82%)	1 (3.57%)	0 (0.00%)	1.000
**Rash**	7 (12.73%)	0 (0.00%)	7 (25.93%)	0.004	1 (1.82%)	0 (0.00%)	1 (3.70%)	0.491
**Hypothyroidism**	10 (18.18%)	0 (0.00%)	10 (37.04%)	<0.001	0 (0.00%)	0 (0.00%)	0 (0.00%)	–
**Intestinal fistula**	3 (5.45%)	1 (3.57%)	2 (7.41%)	0.611	2 (3.64%)	1 (3.57%)	1 (3.70%)	1.000
**Decreased appetite**	13 (23.64%)	9 (32.14%)	4 (14.81%)	0.130	1 (1.82%)	1 (3.57%)	0 (0.00%)	1.000
**Fatigue**	12 (21.82%)	6 (21.43%)	6 (22.22%)	0.943	1 (1.82%)	0 (0.00%)	1 (3.70%)	0.491
**Constipation**	2 (3.64%)	1 (3.57%)	1 (3.70%)	>0.999	0 (0.00%)	0 (0.00%)	0 (0.00%)	–
**Proteinuria**	12 (21.82%)	2 (7.14%)	10 (37.04%)	0.010	1 (1.82%)	1 (3.57%)	0 (0.00%)	1.000
**Diarrhea**	12 (21.82%)	5 (17.86%)	7 (25.93%)	0.469	1 (1.82%)	0 (0.00%)	1 (3.70%)	0.491
**Palmar-plantar erythrodysesthesia syndrome**	5 (9.09%)	0 (0.00%)	5 (18.52%)	0.023	1 (1.82%)	0 (0.00%)	1 (3.70%)	0.491
**Immune-related adverse events**								
**Immune-related pneumonitis**	1 (1.82%)	–	1 (3.70%)	–	0 (0.00%)	–	0 (0.00%)	–
**Immune-related myocarditis**	1 (1.82%)	–	1 (3.70%)	–	0 (0.00%)	–	0 (0.00%)	–
**Radiation therapy-related adverse events**	5 (9.09%)	5 (17.86%)	–	–	0 (0.00%)	0 (0.00%)	–	–

ICIs, immune checkpoint inhibitors; TKIs, tyrosine kinase inhibitors. Two-sided P values were calculated using Pearson’s chi-square test or Fisher’s exact test, depending on the distribution and sparsity of the data. A dash indicates that no statistical comparison was performed.

## Discussion

To the best of our knowledge, this is the first study evaluating the combination of ICIs and TKIs in the treatment of PIK3CA-mutated R/M CC. By analyzing two prospective clinical trials and retrospectively collecting clinical and pathological data from patients receiving Non-ICI/TKI conventional treatments at Fujian Cancer Hospital, we compared the safety and efficacy of the ICIs combined with TKIs group with a physician’s-choice non-ICI/TKI conventional treatment cohort in PIK3CA-mutated R/M CC. Our findings demonstrate that the combination therapy group showed a significant advantage in extending PFS compared to the Non-ICI/TKI conventional treatment group (P = 0.03), while maintaining an acceptable safety profile.

The comparator cohort in this study should be interpreted as a physician’s-choice non-ICI/TKI conventional treatment cohort rather than a homogeneous standard-of-care control group. In real-world clinical practice, patients with previously treated R/M CC often receive individualized treatment strategies according to disease status, prior treatment history, performance status, organ function, treatment availability, and physician judgment. Therefore, the comparator cohort included heterogeneous non-ICI/TKI regimens, such as radiotherapy, chemotherapy, targeted therapy, concurrent chemoradiotherapy, targeted therapy combined with radiotherapy or chemotherapy, and other individualized treatments. Although this heterogeneity limits direct comparison with a standardized control arm, it reflects real-world treatment patterns in a patient population for whom a single universally accepted treatment strategy remains limited.

PIK3CA is one of the most frequently altered genes in cervical cancer [11,12], and its activation promotes tumor-cell proliferation, survival, migration, and treatment resistance through the PI3K/Akt/mTOR signaling pathway [15,16]. The clinical activity observed in this study may reflect the complementary effects of immune checkpoint blockade and anti-angiogenic multikinase inhibition. ICIs enhance antitumor T-cell activity, whereas anlotinib and apatinib primarily inhibit angiogenesis-related receptor tyrosine kinases [17], thereby promoting vascular normalization and modulating the tumor immune microenvironment [18,19]. The present study therefore provides an exploratory evaluation of ICI-TKI combination therapy in patients with PIK3CA-altered cervical cancer, rather than an evaluation of a PIK3CA-directed treatment. Because the analysis was restricted to patients with PIK3CA alterations, these findings describe treatment outcomes within this molecularly defined population. Future studies including a PIK3CA-wild-type comparator are needed to determine whether PIK3CA alteration predicts benefit from ICI-TKI therapy.

Several clinical studies have demonstrated the efficacy of immunotherapy in R/M CC; however, the results have been suboptimal, and effective biomarkers are lacking. In one study, pembrolizumab treatment for R/M CC resulted in an ORR of 14.3%, a DCR of 30.6%, and a median PFS of only 2.1 months [5,20]. Numerous clinical trials have shown the effectiveness of combining immunotherapy with anti-angiogenic drugs, which help normalize the aberrant tumor vasculature, increase immune effector T-cell infiltration, and promote the distribution of T-cells within the tumor, effectively transforming the immune-suppressive tumor microenvironment into an immune-boosting one [10]. Our previous prospective studies and the CLAP clinical trial have confirmed that combining ICIs with TKIs is both safe and effective for the treatment of R/M CC [11,12]. Our study findings indicate that, compared to conventional therapy, ICI-TKI combination therapy showed encouraging clinical activity in patients with PIK3CA-altered R/M cervical cancer, with an ORR of 85.19% (95% CI, 67.52%−94.08%) and a DCR of 88.89% (95% CI, 71.94%−96.15%). These findings support further evaluation of this treatment strategy in prospective studies involving both PIK3CA-altered and PIK3CA-wild-type populations.

The safety profile observed in our cohort was generally consistent with previous reports. At least one grade ≥3 treatment-related adverse event occurred in 12 of 27 patients (44.44%) in the ICIs plus TKIs group and 12 of 28 patients (42.86%) in the Non-ICI/TKI conventional treatment group, with no significant between-group difference (P = 1.000). Anemia was the most common adverse event in the ICIs plus TKIs group (55.56%), whereas anemia and leukopenia were jointly the most common adverse events in the Non-ICI/TKI conventional treatment group (53.57% each). Proteinuria was significantly more frequent in the ICIs plus TKIs group than in the Non-ICI/TKI conventional treatment group (37.04% vs. 7.14%; P = 0.010). The incidence of hypertension was also significantly higher in the ICIs plus TKIs group (48.15% vs. 3.57%; P < 0.001), consistent with the known toxicity profile of TKIs. One patient developed immune-related pneumonitis and one developed immune-related myocarditis in the ICIs plus TKIs group; neither event was grade ≥3. No grade ≥3 immune-related adverse events or treatment-related deaths were reported. These findings emphasize the importance of appropriate patient selection and close monitoring of adverse events during combination therapy.

Although PD-L1 expression has been shown to predict responses to immunotherapy [21], there is still a lack of effective biomarkers for PIK3CA-mutated cervical cancer. Our study provides robust evidence for the application of ICIs combined with TKIs therapy in PIK3CA-mutated R/M CC. Previous studies have explored the efficacy of ICIs in R/M CC, but studies specifically focusing on PIK3CA mutations are still relatively scarce [3,5]. Our results further support the potential of this therapeutic strategy for such patients and provide an important reference for future clinical trials.

However, this study has several limitations. First, this study combined secondary analyses of two prospective clinical trials with a retrospective real-world cohort; therefore, inherent selection bias and information bias may exist. Second, although most baseline characteristics were comparable between the two groups, lung metastases and the number of previous systemic therapies were significantly imbalanced. These factors are clinically relevant and may confound treatment response and survival outcomes. Because of the limited sample size and the small number of events, we did not perform multivariable adjusted analyses. The observed differences in ORR and PFS should be interpreted as exploratory findings, and prospective studies with larger sample sizes and predefined control regimens are needed to validate these results. Third, although we observed the clinical effectiveness of ICIs combined with TKIs, the underlying mechanisms remain incompletely understood. We are currently investigating the molecular mechanisms of ICIs combined with TKIs therapy in PIK3CA-mutated R/M CC and potential biomarkers. Lastly, due to the limited number of patients in our cohort, we were unable to perform subgroup analyses based on different PIK3CA mutation sites. Given the small sample (N = 55) and limited numbers of events (38 PFS events and 30 OS events), a multivariable model including treatment group, lung metastases, and number of previous systemic therapies would be vulnerable to overfitting, sparse-data bias, and imprecise estimates. We therefore retained treatment-group-only univariable models as exploratory analyses. The response and survival differences should be interpreted as unadjusted associations rather than causal treatment effects, because the baseline imbalances may confound the results; adjusted analyses in a larger, prospectively specified cohort are needed.

In this exploratory analysis of patients with PIK3CA-altered R/M cervical cancer, ICIs plus TKIs were associated with higher response rates and longer PFS than physician’s-choice non-ICI/TKI treatments, with a manageable safety profile. Prospective studies including both PIK3CA-altered and PIK3CA-wild-type patients are warranted to confirm these findings and clarify the potential predictive value of PIK3CA alteration.

## Supporting information

S1 Data De-identified individual-level raw data underlying the analyses presented in this study. (XLSX)

S1 Fig The progression-free survival (PFS) (A) and overall survival (OS) (B) survival curves comparing the immune checkpoint inhibitors (ICI) plus Anlotinib group with the ICI plus Apatinib group. (TIF)

S1 Table Treatment efficacy between ICI plus Anlotinib group and ICI plus Apatinib group. Supplementary Material De-identified individual-level source data.
(DOCX)
