# Supplementary material for: Efficacy and safety of immune checkpoint inhibitors combined with TKIs for recurrent or metastatic cervical cancer with PIK3CA mutation
Source: PLoS One. 2026 Sep 29;21(9):e0350571. doi: 10.1371/journal.pone.0350571 (PMC13623110; doi:10.1371/journal.pone.0350571)
Supplement: Supplementary file 1 [file pone.0350571.s003.docx]

Table S1: Treatment efficacy between ICI plus Anlotinib group and ICI plus Apatinib group.

| **Efficacy** | **Group** | | | **p-value** |
| --- | --- | --- | --- | --- |
|  | **Overall**  **(N = 27)** | **ICI plus Anlotinib**  **(N = 13)** | **ICI plus Apatinib**  **(N = 14)** |  |
| **Response Evaluation** |  |  |  | 0.096 |
| PR | 22 (81.48%) | 9 (69.23%) | 13 (92.86%) |  |
| CR | 1 (3.70%) | 1 (7.69%) | 0 (0.00%) |  |
| PD | 2 (7.41%) | 2 (15.38%) | 0 (0.00%) |  |
| SD | 1 (3.70%) | 1 (7.69%) | 0 (0.00%) |  |
| Unknown | 1(3.70%) | 0(0.00%) | 1(7.14%) |  |
| **ORR** | 23 (85.19%) | 10 (76.92%) | 13 (92.86%) | 0.326 |
| **DCR** | 24 (88.89%) | 11 (84.62%) | 13 (92.86%) | 0.596 |
| CR: Complete Response; PR: Partial Response; SD: Stable Disease; PD: Progressive Disease; ORR: Objective Response Rate; DCR: Disease Control Rate; ICI: Immune Checkpoint Inhibitors. P-values were calculated using Fisher’s exact test because of the small sample size. | | | | |
